# RhoA phosphorylation mediated by Rho/RhoA-associated kinase pathway improves the anti-freezing potentiality of *murine* hatched and diapaused blastocysts

**DOI:** 10.1038/s41598-017-07066-2

**Published:** 2017-07-27

**Authors:** Meichao Gu, Hemin Ni, Xihui Sheng, Alfredo Pauciullo, Yunhai Liu, Yong Guo

**Affiliations:** 1 College of Animal Science and Technology, Beijing University of Agriculture, Beijing, China; 2 Department of Agricultural, Forest and Food Sciences, University of Torino, Grugliasco, Italy

## Abstract

Embryonic cryopreservation has a relatively low survival rate because of cytoskeletal damage. However, molecular anti-freezing mechanisms have been largely unexplored. This study investigated the significance of RhoA, involved in embryonic development, and the Rho/RhoA-associated kinase (ROCK) signalling pathway in cryopreservation. The anti-freezing mechanism in murine dormant embryos, compared with normal blastocysts, was assessed by combining molecular, physiological and pharmacological approaches. Real-time PCR and western blotting experiments showed high RhoA expression in cryo-dormant and dormant embryos. RhoA GTPases were overexpressed on the surface of trophectoderm cells in dormant embryos. Treatment with Y-27632, a ROCK antagonist, decreased survival of both normal and dormant blastocysts, while recombinant RhoA protein remarkably increased survival, after freeze–thawing, of normal hatched blastocysts. Our findings elucidated the molecular mechanism of anti-freezing, involving RhoA phosphorylation, meditated by the Rho/ROCK signalling pathway, in hatched and diapaused murine blastocysts. In addition, evidence for a potentially protective additive suggests a new method for improving the anti-freezing potential of mammalian embryos, without protecting the zona pellucida.

## Introduction

It is widely known that mammalian delayed implantation is of great importance in reproductive biology. During entry and maintenance of diapause, cell cycle progression is arrested by p21 protein family and is restrained at G1^1^. For instance, in mice and rats, ovariectomy before the presumed oestrogen surge, on the morning of day 4 of pregnancy, results in failure of implantation and initiates a dormancy state in blastocysts^2^.

Previous studies showed that dormant mouse embryos have higher survival rates than normal embryos after cryopreservation^3^. Generally, expansion ability after freezing-thawing procedure is reckoned as an indicator of embryonic survival status^4^. Although cryopreservation of mammalian oocytes is a routine technique used worldwide, it is still crucial to decrease cryo-damage of cells during cryopreservation steps for efficient survival of embryos or oocytes. Cryo-injury influences embryonic development, even interfering with the cytokinesis and receptor-mediated signal transduction^5–8^.

RhoA is a small guanosine triphosphatase (GTPase) protein from the Rho family and it belongs to Ras homologue gene family. RhoA was reported to be involved in complex cellular processes, including cell motility, cell adhesion and chromosome inheritance^9^. RhoA is a Rho family regulator of cytokinesis in dividing embryos^10^, whereas Rho-associated protein kinase (ROCK) is a key downstream effector of RhoA. It was reported that the embryonic development is dependent on RhoA in *Xenopus* and *Drosophila melanogaster* ^11–13^. In addition, blocking of ROCK prevented early cleavage development in early zebrafish and mouse embryos^14^. RhoA also plays a pivotal role in G1 cell cycle progression, primarily by regulating expression of cyclin D1 and cyclin-dependent kinase inhibitors (p21 and p27). On one hand, RhoA facilitates the entry into S phase by degradation of the cyclin-dependent kinase inhibitor p27^kip1^ ^15^. On the other hand, it suppresses p21 levels that block G1. As a binary molecular “switch”, RhoA GTP can be catalyzed by guanine nucleotide exchange factors (GEFs), which phosphorylate the GDP in GTP^10^. Y-27632 is a specific Rho-kinase inhibitor, which showed excellent selectivity against RhoA-associated kinases (ROK/ROCK)^16, 17^.

Gene expression and proteomic analysis of normal hatched, dormant and reactivated blastocysts have indicated differentially expressed RhoA. In fact, RhoA showed higher expression in cryo-dormant blastocysts compared to normal hatched^18^. Proteomic analysis of dormant and reactivated blastocysts revealed higher levels of RhoA protein in active blastocysts than in the dormant ones^19^.

Together, such evidences suggest that RhoA gene could provide novel insights into the study of the anti-freezing potentiality of dormant blastocysts in mice. Furthermore, the connections between the role of RhoA and cryopreservation of mammalian blastocysts have never been elucidated. Therefore, in the present study, we have applied both molecular and cellular approaches to contribute to a more detailed knowledge on the role of RhoA in cryopreservation.

## Results

### RhoA Is Overexpressed in Dormant Blastocysts Compared to Normal Ones

**Figure 1:**
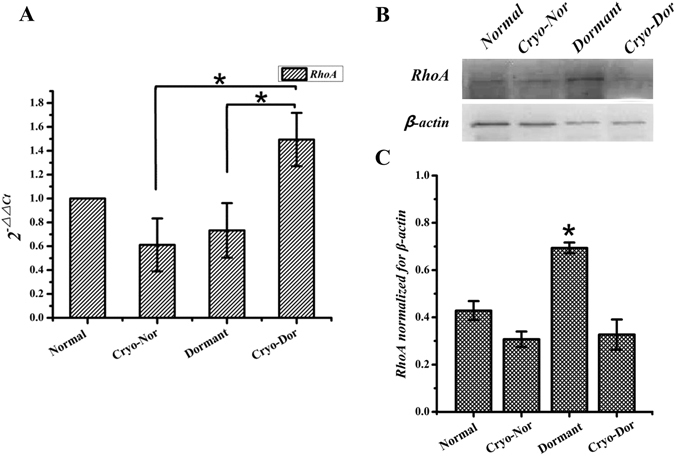
RhoA gene expression and Western blot analysis (cropped blots) in dormant and normally hatched embryos. (**A**) RhoA transcription was analysed in various blastocyst groups. Concentrations of RhoA mRNA were normalised to those of *GAPDH*. Values expressed as means ± SEM, *p < 0.05. (**B** and **C**) Western blot profiles of total RhoA normalized with β-actin. Values expressed as mean ± SEM, *p < 0.05.

Based on previous findings^18^, we investigated the expression of RhoA in dormant blastocysts by Real Time-PCR and Western blot. The expression of RhoA mRNA after freezing-thawing step showed a dramatic up-regulation in cryo-dormant embryos compared to cryo-normal and dormant blastocysts (Fig. 1A). Conversely, Western blot results showed upregulation of total RhoA protein in dormant embryos, compared with in normal and cryo-dormant embryos (Fig. 1B).

### RhoA GTPases Are Up-Regulated in Dormant Blastocysts

**Figure 2:**
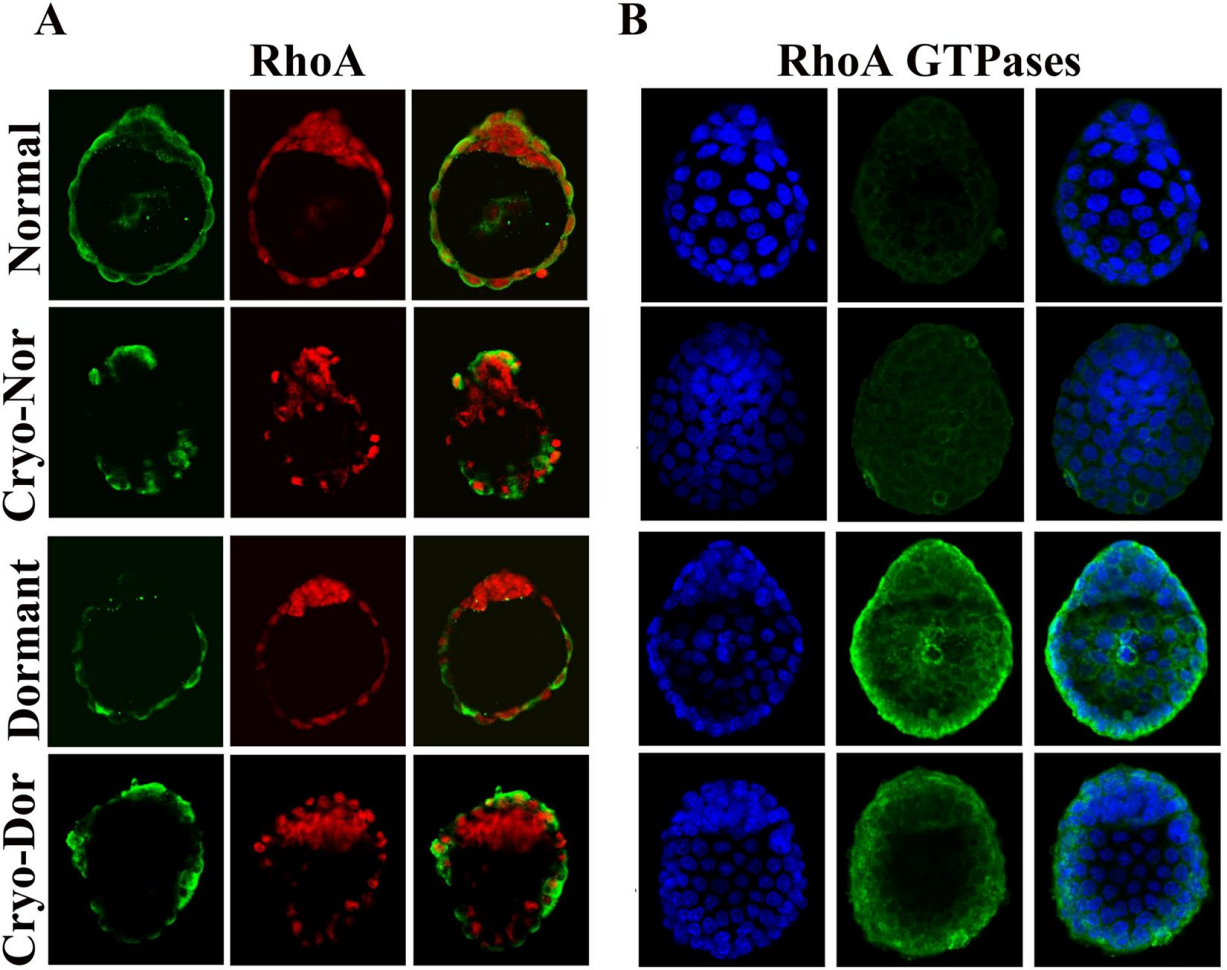
Localisation of total RhoA and phospho-RhoA in mouse embryos (10 × 20 magnification). (**A**) Images show RhoA antigen labelled in green, propidium iodide labeled nuclei in red, and the merged images. (**B**) images showing phospho-RhoA antigen labelled in green, Hoechst 33342 labelled nuclei in blue, and the merged images.

Since phosphorylation is one of the main post-translational protein modifications, we investigated phosphorylated RhoA levels. RhoA and phosphorylation of RhoA (RhoA GTPases) were analysed in embryos using confocal microscopy. The results revealed that the RhoA protein was present only in trophectoderm cells (TE) (Fig. 2A), whereas phosphorylated RhoA (RhoA GTPases) levels were higher in dormant and cryo-dormant blastocysts than in normal and cryo-normal blastocysts (Fig. 2B). In addition, both the total and GTP-bounded (active) RhoA GTPases were up-regulated in TE cells of dormant embryos. Furthermore, differences among RhoA GTPases (Fig. 2B) were remarkable compared with total RhoA levels in embryos (Fig. 2A).

### RhoA Down-Regulation by Blocking the Rho/ROCK Pathway Decreased Embryos Survival Rates

Since RhoA expression analysis showed over-expression in dormant blastocysts, we cultured dormant embryos *in-vitro* with the specific inhibitor Y-27632, to supress the RhoA-associated kinase (Rho/ROCK) pathway and assess effects on their survival rates after freezing-thawing. The results are reported in Table 1. The supplementation of the medium with Y-27632 significantly decreased the survival rate of both normal and dormant blastocysts (p < 0.01). No significant differences were observed between two control groups for 0 h and 4 h of *in-vitro* culture.

**Table 1:** Survival rates after freezing-thawing of blastocysts, co-cultured with recombinant protein RhoA and its inhibitor, as indicated.

Different Treatments	Normal Embryos (%)	Dormant Embryos (%)
Control (0 h)	48.26 ± 8.34	70.10 ± 6.40
Control (4 h)	45.01 ± 10.06	64.21 ± 12.02
Y-27632 (4 h)	0.00 ± 0.00^**^	0.00 ± 0.00^**^
Recombinant Protein (4 h)	63.31 ± 5.00^*^	78.00 ± 6.01
Recombinant Protein** + **Y-27632 (4 h)	2.20 ± 1.21^**^	0.00 ± 0.00^**^

Note: The data are presented as the means ± SEM. **p < 0.01 was considered remarkable statistically significant in same column; *p < 0.05 was considered statistically significant in same column. 0 h: directly frozen without *in-vitro* culture. 4 h: *in-vitro* culture for 4 hours before freezing.

### Recombinant RhoA Protein Improved Survival in Cryopreserved Normal Blastocysts, but Did not Prevent Damage Caused by Y-27632 in Embryos

The survival rate after freezing-thawing of blastocysts co-cultured with recombinant RhoA protein was significantly higher (p < 0.05) than that of control hatched blastocysts (Table 1). In particular, we found that the recombinant RhoA significantly improved the survival rate of normal blastocysts, but did not affect dormant blastocysts. Conversely, the survival rate of blastocysts was decreased when the recombinant protein was used in combination with the inhibitor, showing the dramatic effects of Y-27632 on both normal and dormant embryos (p < 0.01).

## Discussions

In our previous study, Gu *et al*.^3^ found that dormant blastocysts had a significantly higher survival rate after freezing-thawing than hatched embryos. In a further analysis, Zhang *et al*.^18^ also showed that the total RhoA levels in dormant blastocysts were significantly higher than in normally hatched embryos^18^. Therefore, we further investigated the functional role of RhoA in embryo cryopreservation, using various biological approaches, including different medium formulations for *in-vitro* culture, Real Time PCR, Western blot and confocal microscopy.

The two cryo-normal groups showed down-regulation compared to the normal groups both in Real-Time PCR and Western blot analysis. It is known that cryopreservation affects transcripts stability making some of them prone to degradation. For instance, in human sperm the cryopreservation affects mRNA-protein interaction making mRNA molecules more susceptible to degradation^20^. In addition, cryopreservation was reported to produce a decrease in most of gene transcripts and the up-regulation of heat shock protein. This effect was caused by freezing/thawing rather than exposure to cryoprotectants^21^. This is consistent with our results.

In addition, transcript expression and Western blot analysis showed that RhoA is over-expressed in cryo-dormant group compared to dormant group. We proposed that the up-regulation of RhoA in cryo-dormant blastocysts after thawing contributed to a higher survival rate compared to the normal ones. We expected a consistent trend in the dormant groups, however the results of the Western blot analysis showed an inverse trend. These results evidenced that RhoA expression and translation in blastocysts were inversely correlated. Greenbaum *et al*.^22^ have extensively reported the reason for a poor correlation between the level of mRNA and protein. First, there are many complicated and varied post-transcriptional mechanisms involved in turning RhoA mRNA into protein, including phosphorylation (e.g AMPk); second, RhoA protein may differ substantially in their *in vivo* half-life; third, there is a significant amount of error and noise in both protein and mRNA experiments that limit our ability to get a clear picture. In addition, it should be taken into account that sensitivity of Western blot analysis is affected by the specificity of antibody reactions. In our study, although the reaction was optimised for antibody concentration and incubation time, the anti-RhoA antibody still produced strong background staining, compared with the *β*-actin control. This may explain the different results obtained with Western blot and Real-time analyses. Nevertheless, RhoA gene expression in dormant blastocysts was higher than normal ones.

RhoA is widely recognised to play important roles in regulating the cytoskeleton and cell division, and it has also been identified as a key mediator of membrane ruffling and lamellae formation^23^. Therefore, we applied immune-histochemical staining to evaluate the distribution of RhoA protein on different types of blastocysts. Confocal microscopy revealed the exclusive presence of RhoA signal in the embryonic TE cells. In addition, a further experiment demonstrated differences in levels of active phosphorylated RhoA (RhoA GTPases), showing clearly higher staining in dormant and cryo-dormant blastocysts than in normal and cryo-normal hatched ones. These differences among groups strongly supported our hypothesis that the mechanism of anti-freezing in embryos is associated with the expression of RhoA gene or its post-translational regulation (phosphorylation/desphosphorylation) of RhoA. Therefore, investigating RhoA GTPases provided novel insights into the mechanism of anti-freezing in dormant mouse blastocysts.

Recently, Zhang *et al*.^24^ showed that, in porcine oocyte meiosis, disruption of RhoA activity and the knock-down of RhoA gene expression caused failure of polar body emission. In mice, the small GTPases RhoA might be a potential upstream regulator of formin-like protein 1 (FMNL1), which affects spindle organisation and action in oocytes polar body extrusion^25^. Therefore, the RhoA is crucial for embryonic morphogenesis. In our previous study, using transmission electron microscopy, we observed the ultra-structure of dormant blastocyst TE cells^3^. We proposed that the role of RhoA was connected to the noncanonical Wnt pathway, a signalling process independent from *β*-catenin regulation^26^. Down-regulation of RhoA GTPases led to cytoskeletal reorganisation and disassembly of adherens junctions, which destabilize epithelial TE cells to blastocyst-uterine attachment^27–29^. Briefly, the Wnt-RhoA signalling pathway ensures blastocyst competency to implantation and the small GTPases of Rho family are potential mediators of the Wnt pathway. Rho acts downstream, with Rho-associated kinase (ROCK) acting as one effector^30^. Despite the existence of indistinct signalling networks, the Rho family GTPases and their effectors remain the most obvious link between noncanonical Wnt signalling and the cytoskeleton.

To verify the role of RhoA kinase (post-translational regulation) on blastocyst survival rates after freezing–thawing, three different *in vitro* culture groups were examined. No blastocysts survived when Y-27632 was added alone, and survival rates were significantly decreased in the presence of a combination of recombinant RhoA and Y-27632, in both normal and dormant blastocysts (p < 0.01). These results suggest that the inhibition of the RhoA/ROCK pathway induced by Y-27632 in mouse blastocysts was non-reversible. Therefore, the specific RhoA inhibitor restrained the survival rate after cryopreservation by blocking the Rho/ROCK signalling pathway.

Supplementation of the medium with recombinant RhoA protein significantly increased the survival rate of normal blastocysts, compared with the controls (p < 0.05), but did not affect dormant blastocysts. However, dormant blastocysts had higher survival rates than the normal blastocysts, with and without recombinant RhoA treatment. Therefore, phosphorylation of recombinant RhoA could only improve the anti-freezing characteristics of normal murine hatched embryos, mediated by the Rho/ROCK signalling pathway, whereas it seems to have not the same effect on dormant embryos. It is likely that the dormant embryos already have a high level of phosphorylated RhoA, so that their own anti-freezing potentiality is not improved by adding further recombinant protein.

**Figure 3:**
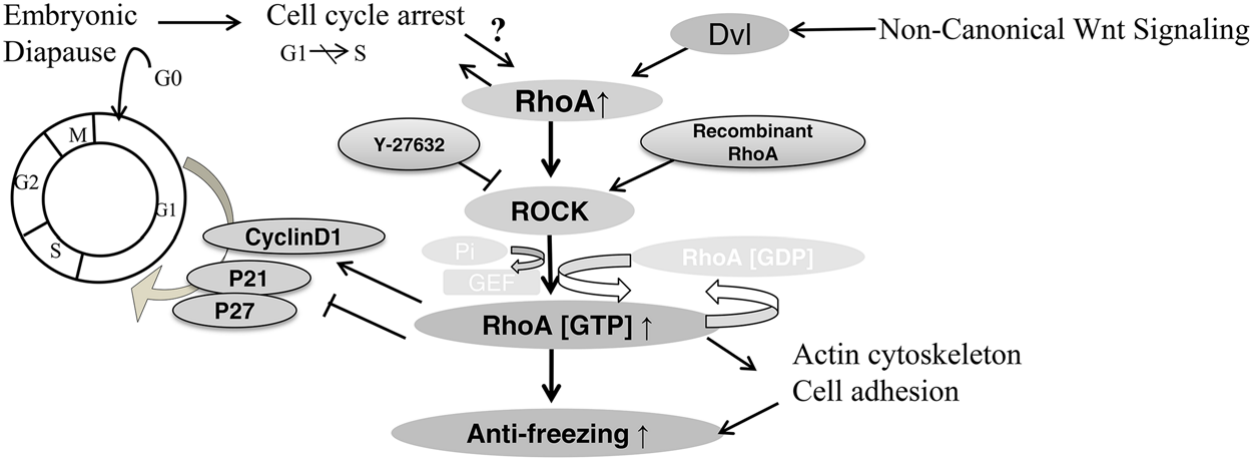
Schematic diagram showing the RhoA pathway in cryopreservation of dormant murine embryos. The RhoA/ROCK pathway may improve the anti-freezing capacity of embryos, through two signalling pathways: embryonic diapause and non-canonical Wnt signalling.

RhoA plays a pivotal role in G1 cell cycle progression, primarily through regulation of expression of cyclin D1 and cyclin-dependent kinase inhibitors (p21 and p27). These regulation pathways activate protein kinases, which subsequently modulate transcription factor activity. Entry and maintenance of diapause in dormant blastocysts result from expression of cell cycle inhibitors of the p21 protein family, which interfere with cyclin E-cdk2 complex formation necessary for progression through G1. Therefore, it is possible that basal RhoA levels in dormant blastocysts can activate p21 expression and that cell cycle blockage is already responsible for increasing cryopreservation in the dormant blastocysts. Unlike the normal blastocysts, the dormant ones do not undergo RhoA-induced cell cycle blockage and p21 expression^31^. Consequently, we suggested two possible pathways to explain why murine dormant blastocysts have higher survival rates than normal ones. In addition, the Rho/ROCK pathway improved the anti-freezing capacity of mouse blastocysts (Fig. 3).

Embryonic diapause occurs because of suppression of cell proliferation at the blastocysts stage. During regulation of the cell cycle in diapause, different proximal signals and cellular factors coordinate with uterine factors to arrest the developmental stage. During this phase, traces of ovarian oestrogens can induce mitotic restart of embryos^1^. Additionally, the metabolic activities of dormant blastocysts are different from those of the activated blastocysts^19^. Dormant embryos before freezing are characterized by harsh living conditions and their energy metabolism is reduced to a “basal level”, which allows them to have better anti-freezing properties than normally hatched blastocysts^3^. However, it remains unknown which event triggers the embryonic anti-freezing mechanisms during cryopreservation. Combining molecular, physiological and pharmacological approaches, we showed that the Rho/ROCK signalling pathway plays key roles in murine blastocysts cryopreservation. Similarly, we demonstrated failure of the blastocysts survival when the Rho/ROCK signalling pathway was blocked by Y-27632. This is consistent with the finding that RhoA down-regulation destabilised the edges of TE cells and weakened the cytoskeleton in embryos^29^. Our results suggest that up-regulation of RhoA and RhoA GTPases in dormant embryos could reinforce the cytoskeleton and, therefore, improve the anti-freezing potentiality of *murine* hatched and dormant blastocysts.

These findings increased knowledge of mammalian cryopreservation mechanisms, suggesting that the Rho/ROCK signalling pathway can be one of the main key points to determine the fate of the embryos during the cryopreservation. Furthermore, the supplementation with recombinant RhoA protein could increase the survival of blastocysts, indicating that it is a protective anti-freezing additive. However, this aspect has to be further clarified, whereas RhoA phosphorylation, mediated by the Rho/ROCK pathway, might improve the anti-freezing potential of murine hatched and delayed blastocysts.

## Materials and Methods

### Ethical approval

The Ethics Committee of Beijing University of Agriculture approved the study (Permit Number 2012-0611) and all the methods were carried out in accordance with the approved guidelines.

Mice were bought from Beijing Vital River Laboratory Animal Technology Co., Ltd. (SCXK Beijing 2012-0001) and housed in the Institutional Animal Care Facility of Beijing University of Agriculture (SYXK Beijing 2010-0003) according to institutional guidelines for laboratory. Procedures were conducted in accordance with the ethical standards of Ministry of Science and Technology of China applied by the Institutional Animal Care Committee of the Beijing University of Agriculture. No endangered or protected species were involved in this study.

### Animal Models and Blastocysts

A total of 200 female ICR mice, 6–8 week old, were used for the present study. Superovulation was induced by intraperitoneal injection of 10 IU pregnant mare serum gonadotropin (PMSG) and human chorionic gonadotropin (hCG), given 46–48 h before mating with 12 week old ICR males. Mating was confirmed by the presence of a copulation plug in the morning and, then, 10 IU of anti-pregnant mare’s serum gonadotropin (A-PMSG) was administered to the females by injection^32^. Mice were bilaterally ovariectomised in the morning (08.00–09.00 h) of day 4, and then they were treated daily with 0.2 g progesterone, prepared in 10 ml sesame oil until day 7 to induce delayed implantation. A total of 800 dormant blastocysts were collected on day 8. In addition, 800 normally hatched blastocysts were collected from control intact mice on day 5.

### Freezing-thawing of Embryos

Harvested mouse blastocysts were washed twice with PBS (Sigma-Aldrich, St. louis, MO, USA) and then washed twice in frozen fluid (ICPbio Reproduction; 101129, Auckland, New Zeland) to perform the freezing step. Briefly, the mouse embryos were aspirated into a 0.25 ml plastic straw. The cooling rate of the freezing apparatus was set up to −1 °C/min until samples reached a temperature of −5 °C. Within 10 min, using tweezers, the straws containing the embryos were cooled down, showing an ice layer on the top within 3–5 s. Then, frozen tubes were stored into liquid nitrogen until the cooling rate was changed to −0.3 °C/min until the temperature reached −35 °C and then stored in liquid nitrogen.

To thaw samples, the frozen tubes were removed from liquid nitrogen, gently shacked at room temperature for 5–10 s, and moved into a 35 °C water bath for 10 s. Afterwards, the blastocysts were quickly transferred to a glass dish containing 1 M sucrose solution.

### Quantitative Real Time PCR Analysis (Q-PCR)

Comparative RhoA gene expression was accomplished in 4 groups of embryos, normal hatched and dormant embryos, each before and after the cryopreservation treatment. A total of 100 blastocysts in each group were used to extract total RNA. Reverse transcription reactions were performed using oligo dT_18_. Real-time PCR was performed using the CFX96 Touch Real-Time PCR Detection SYBR Green Supermix (BioRad, Hercules, CA, USA). The following RhoA gene specific primers were used for real-time amplification: *forward* 5′-ACT CGG AGT CCT CGC CTT GA-3′ and *reverse* 5′-AAG CTC CAT CAC CAA CAA TCA C-3′ (GenBank accession number NM_ 016802.5). *GAPDH* was chosen as the housekeeping gene (GenBank accession number NM_ 008084.3) and amplified with the following primers: *forward* 5′-TGG CAA AGT GGA GAT TGT TGC C-3′ and *reverse* 5′-AAG ATG GTA ATA AAC TTC CCG-3′. Samples were run in triplicate, and the relative gene expression was calculated using the comparative threshold cycle (Ct) and normalised to expression of *GAPDH* (ΔCt). Results are expressed as fold change relative to the mean.

### Western Blot Analysis of Blastocysts

A total of 200 blastocysts were collected for each treatment condition. Samples were boiled in 5X loading buffer for 10 min and rapidly transferred to ice. Protein samples were electrophoresed using 10% SDS-polyacrylamide gels and bands were transferred to a PVDF membrane (Millipore, MA, USA). The membranes were blocked for 2 h in PBS with 0.1% Tween-20 (PBST) containing 5% of skim milk and then were incubated overnight at 4 °C with primary antibodies (1:1000 dilution). A mouse monoclonal *β*-actin antibody (Sigma-Aldrich) at 1:2000 dilution was used to control for sample loading. After washing 3 times with PBST, the membranes were incubated at 37 °C for 1 h with a donkey anti-rabbit immunoglobulin (IgG) secondary antibody (1:10000 dilution; Zhongshan Golden Bridge Biotechnology Co., Ltd, China). Protein bands were visualised using the ECL Plus Western Blotting Detection System Tanon-5500 (BioRad). To quantify western blot results, band intensity values were determined using Image J software (National Institute of Health; Bethesda, MD, USA).

### Immunofluorescence Staining and Confocal Microscopy

For observing the distribution of total RhoA and the active RhoA-GTP, immunofluorescence staining of blastocysts was performed as described by Wang *et al*.^33^ using an anti-RhoA antibody (ab86279, Abcam) and an anti-active RhoA antibody (26904, New East Biosciences, King of Prussia, PA, USA). Slides were observed by confocal scanning laser microscope (LSM 710 model; Zeiss, Jena, Germany) and images analysed with Zeiss LSM image browsing software.

### Evaluation of Effects of Y-27632 and Recombinant RhoA on Embryonic Viability after Cryopreservation

To determine the effects of Y-27632 on blastocysts, the normal and dormant embryos were cultured in Whitten’s solution supplemented with various concentrations of Y-27632 (0, 1, 10, 20, 100 μM; Sigma-Aldrich) for 4 h. A Y-27632 concentration of 20 μM was determined to be optimal. Recombined RhoA protein (Sino Biological Inc., Beijing, China) was diluted with the culture medium for optimal storage (100 μg/μl). Final concentration (1, 10, 20, 50 μg/μl) was tested in culture medium with embryos. A concentration of 10 μg/μl was then used to culture blastocysts for 4 h. The embryos were cultured in 50 μl drops of fresh pre-equilibrated culture medium at 37 °C in 5% CO_2_. Three replicate experiments were conducted, treating and evaluating a total of 360 embryos.

After freezing-thawing, the embryos were washed in Whitten’s solution and cultured in the same medium, covered with mineral oil at 37 °C in 5% CO_2_ and saturated humidity. Embryos showing re-expansion in post-thawing/warming cultures were considered to be alive. The percentages of embryos showing re-expansion were determined at 4 h intervals during 24 h of additional culture after thawing/warming. In various treatment groups, the percentage of re-expanded blastocysts, determined at each time point, were used to calculate the survival rates^4^.

### Statistical Analysis

Data are presented as means ± SEM. Statistical comparisons of data were performed using analysis of variance (ANOVA), followed by Student-Newman-Keuls test, calculated with SPSS software (IBM, New York, USA). P-values lower than 0.05 were considered significant.
